# Fluorescent Materials with Excellent Biocompatibility and Their Application in Bio-Sensing, Bio-Imaging

**DOI:** 10.3390/bios13100906

**Published:** 2023-09-26

**Authors:** Meng Zheng, Yalong Wang, Deteng Zhang, Mingqiang Zhu

**Affiliations:** 1 State Key Laboratory of Digital Medical Engineering, School of Biomedical Engineering, Hainan University, Haikou 570228, China; zhengmeng.555@hotmail.com (M.Z.); mqzhu@hainanu.edu.cn (M.Z.); 2 R&D Center of Polymer Materials, Qingdao Haiwan Science and Technology Industry Research Institute Co., Ltd. (HWSTI), Qingdao 266031, China; 3 Institute of Neuroregeneration and Neurorehabilitation, Qingdao University, Qingdao 266071, China; 4 Wuhan National Laboratory for Optoelectronics, School of Optical and Electronic Information, Huazhong University of Science and Technology, Wuhan 430074, China

Fluorescent materials have great potential for use in biomedical applications due to their ease of functionalization and tunable fluorescence color. In the last couple of decades, major progress has been made in the synthesis, characterization, and application of fluorescent materials, along with the development of nano-, bio-based, sensor, imaging and high-performance-material-based technologies, which is believed to contribute to the extension of their applications in bio-sensing, bio-labeling, bio-tracing, bio-imaging, disease diagnosis and therapy, etc. Biocompatibility is the top priority when fluorescent materials are used in bio-fields; a great number of biocompatible fluorescent materials (BFMs) have been obtained, such as organic small-molecule dyes, fluorescent conjugated polymers, metal nanoclusters, rare earth ion-based nanoparticles (e.g., rare-earth-doped upconversion nanoparticles), quantum dots, carbon nanomaterials (e.g., carbon nanotubes and carbon nanodots) and various fluorescent proteins. This Special Issue, “Fluorescent Materials with Excellent Biocompatibility and Their Application in Bio-Sensing, Bio-Imaging”, includes twelve research articles covering biosensors based on ion sensors, the detection of pathological oxidative stress and disease detection [1,2,3,4,5,6,7,8,9,10,11,12], as well as four review articles focused on different types of fluorescent probes and biological applications [13,14,15,16].

As the most abundant small molecular biological thiol, glutathione (GSH) plays an important role in body metabolism. Abnormal GSH levels have been shown to be associated with the dysfunction of specific physiological activities and certain diseases, such as cancer. Thus, the detection of glutathione has become a critical issue. Prof. Pavlova fabricated a new GSH probe, BChl–S–S–NI, based on the second-generation photosensitizer bacteriochlorin e6 (BChl) and a 4-styrylnaphthalimide fluorophore (NI) [1]. They verified that the energy transfer process of BChl–S–S–NI is realized with high efficiency in the conjugated system, leading to the emissions of the fluorophore fragment being quenched. However, in the presence of GSH, the fluorescence is activated, eliminating the possibility of energy transfer due to the cleavage of the molecule into separate functional fragments. Hou’s group reported another GSH probe, CDS-NBD, which could make a distinction between GSH and Cys/Hcy [2]. The CDS-NBD probe could also be successfully studied in the imaging of Cys/Hcy and GSH in vivo. In the review, Prof. Sun focused on the fluorescence detection methods of GSH and discussed the principle of GSH fluorescence sensing [13]. In addition, a general overview of biological applications related to GSH fluorescence sensing was provided. Finally, future opportunities and challenges in GSH fluorescence sensing, especially facilitating disease diagnosis, were discussed. Reactive oxygen species (ROS), including ^1^O_2_, ·OH-, H_2_O_2_, and ·O_2_-, are closely related to many pathophysiological processes in organisms. Excessive hydrogen peroxide led to diseases such as cancer, diabetes, and cardiovascular diseases. Therefore, it is vital to detect ROS for the diagnosis and treatment of diseases. Prof. Peng reported a novel photoswitch aggregation-induced emission (AIE) photosensitizer, 6BrHTI-TPA-OMe, which could photocontrol ^1^O_2_ generation for tumor therapy [4]. Prof. Xu [5] and Prof. Samanta [6] developed fluorescent probes activated by H_2_O_2_ to monitor the content of H_2_O_2_ in cells. Mateo’s group prepared fluorescent nanocomposite hydrogels with high swelling and adsorption capacity that can quantitatively detect the presence of enzyme alkaline phosphatase (ALP) in water samples with a response time of 10 min and a LOD of 21 nm [7]. Prof. Li designed and synthesized a novel aggregation-induced fluorescent probe, PTPA-QM, which showed high affinity for beta-amyloid in brain sections of 5XFAD in mice with classical inflammatory cognitive impairment [8]. This provided a promising tool for the detection of beta-amyloid protein.

Heavy metal or trace element content in the external water environment or in the human body can cause changes in human physiological activities. The intake of heavy metals can affect the central nervous system, vascular system, and blood system, which can thus seriously affect the health of the body. Changes in trace element content in the human body are closely related to many diseases. Therefore, it is very important to monitor the content of heavy metals or trace elements in the external water environment or the human body. Prof. Cao constructed a fluorescent supramolecular system (H-⊃G) for Hg^2+^ detection in 100% water. This supramolecular system has excellent selectivity for Hg^2+^, and the detection limit is 7.17 × 10^−7^ M [9]. Three works reported on biosensors for the detection of Cu^2+^, which are capable of monitoring the amount of Cu^2+^ in cells [10,11,12,13].

Materials used in the construction of biofluorescence sensors need to possess excellent biocompatibility, non-toxicity, and efficient luminescence properties. A summary of different fluorescence sensors is of guiding significance for the design of probes with better performance. Prof. Hsiao reviewed excellent probes based on brightness, light stability, and functionalized fluorescent nanodiamonds (FNDs) [14]. The altered surface chemistry of FNDs and their combination with polymers, metals, and magnetic nanoparticles opens up huge possibilities for drug delivery, diagnostics, and nanomedical and magnetothermal therapy. Prof. Zhang summarized biofluorescence probes of aggregation-induced emission (AIE) materials with the donor–acceptor (D-A) system and hydrogen bonding [15]. Furthermore, they discussed the design concept and potential development trend of biofluorescent probes. This review provides a theoretical framework for the design of high-performance AIE bioluminescence probes, and it can be used to further develop the application of AIE-based bioluminescence probes. Organic semiconductor nanoparticles (OSNs) are used as high-resolution biosensors in modern medicine and biological research. OSNs have been widely used in the detection of biological small molecules, nucleic acid and enzyme levels, as well as vascular imaging, tumor localization, etc. Based on the structure and design strategy of OSNs, this review introduces various functional group OSN biosensors and discusses the design strategies and future development trends of OSN-based biosensors [16].

Fluorescent probe technology has the advantages of simplicity, convenience, high sensitivity, being conducted in real time, and being non-invasive, and it has been widely used to image important biological substances (such as ions, signaling small molecules, and enzymes) in living cells and organisms to explore the physiological and pathological processes of related diseases. This Special Issue covers the preparation of biosensors using organic and inorganic fluorescent materials. These studies have shown that fluorescence sensors show great potential in biomedical applications.

## References

[1] Pavlova M.A., Panchenko P.A., Alekhina E.A., Ignatova A.A., Plyutinskaya A.D., Pankratov A.A., Pritmov D.A., Grin M.A., Feofanov A.V., Fedorova O.A. (2022). A New Glutathione-Cleavable Theranostic for Photodynamic Therapy Based on Bacteriochlorin e and Styrylnaphthalimide Derivatives. Biosensors.

[2] Hou H., Liu Q., Liu X., Fu S., Zhang H., Li S., Chen S., Hou P. (2022). Dual Response Site Fluorescent Probe for Highly Sensitive Detection of Cys/Hcy and GSH In Vivo through Two Different Emission Channels. Biosensors.

[3] Zhang H., Li A.Z., Liu J. (2023). Surfactant-Assisted Label-Free Fluorescent Aptamer Biosensors and Binding Assays. Biosensors.

[4] Wang J., Wei J., Leng Y., Dai Y., Xie C., Zhang Z., Zhu M., Peng X. (2023). Rational Design of High-Performance Hemithioindigo-Based Photoswitchable AIE Photosensitizer and Enabling Reversible Control Singlet Oxygen Generation. Biosensors.

[5] Wang K., Yao T., Xue J., Guo Y., Xu X. (2023). A Novel Fluorescent Probe for the Detection of Hydrogen Peroxide. Biosensors.

[6] Baruah M., Jana A., Pareek N., Singh S., Samanta A. (2023). A Ratiometric Fluorescent Probe for Hypochlorite and Lipid Droplets to Monitor Oxidative Stress. Biosensors.

[7] Alacid Y., Esquembre R., Montilla F., Martínez-Tomé M.J., Mateo C.R. (2023). Fluorescent Nanocomposite Hydrogels Based on Conjugated Polymer Nanoparticles as Platforms for Alkaline Phosphatase Detection. Biosensors.

[8] Fang Y., Wang Q., Xiang C., Liu G., Li J. (2023). A Novel Aggregation-Induced Emission Fluorescent Probe for Detection of β-Amyloid Based on Pyridinyltriphenylamine and Quinoline–Malononitrile. Biosensors.

[9] Jiang X., Wang L., Ran X., Tang H., Cao D. (2022). Green, Efficient Detection and Removal of Hg^2+^ by Water-Soluble Fluorescent Pillar[5]arene Supramolecular Self-Assembly. Biosensors.

[10] Leng X., Wang D., Mi Z., Zhang Y., Yang B., Chen F. (2022). Novel Fluorescence Probe toward Cu^2+^ Based on Fluorescein Derivatives and Its Bioimaging in Cells. Biosensors.

[11] Bai Y., Zhang H., Yang B., Leng X. (2023). Development of a Fluorescein-Based Probe with an “Off–On” Mechanism for Selective Detection of Copper (II) Ions and Its Application in Imaging of Living Cells. Biosensors.

[12] Speghini R., Buscato C., Marcato S., Fortunati I., Baldan B., Ferrante C. (2023). Response of *Coccomyxa cimbrica* sp.nov. to Increasing Doses of Cu(II) as a Function of Time: Comparison between Exposure in a Microfluidic Device or with Standard Protocols. Biosensors.

[13] Sun X., Guo F., Ye Q., Zhou J., Han J., Guo R. (2023). Fluorescent Sensing of Glutathione and Related Bio-Applications. Biosensors.

[14] Qureshi S.A., Hsiao W.W.-W., Hussain L., Aman H., Le T.-N., Rafique M. (2022). Recent Development of Fluorescent Nanodiamonds for Optical Biosensing and Disease Diagnosis. Biosensors.

[15] Wang Z., Ma J., Li C., Zhang H. (2023). Conjugated Aggregation-Induced Fluorescent Materials for Biofluorescent Probes: A Review. Biosensors.

[16] Wang Z., Han D., Wang H., Zheng M., Xu Y., Zhang H. (2023). Organic Semiconducting Nanoparticles for Biosensor: A Review. Biosensors.

